# Single-cell identification by microfluidic-based *in situ* extracting and online mass spectrometric analysis of phospholipids expression†

**DOI:** 10.1039/c9sc05143k

**Published:** 2019-11-11

**Authors:** Qiushi Huang, Sifeng Mao, Mashooq Khan, Weiwei Li, Qiang Zhang, Jin-Ming Lin

**Affiliations:** Department of Chemistry, Beijing Key Laboratory of Microanalytical Methods and Instrumentation, MOE Key Laboratory of Bioorganic Phosphorus Chemistry & Chemical Biology, Tsinghua University Beijing 100084 China jmlin@mail.tsinghua.edu.cn

## Abstract

This work describes a microfluidic system for *in situ* extraction of a single-cell and its phosphatidylcholine analysis through mass spectrometry. This approach uncovered cellular heterogeneity among seemingly identical cells and provided a new platform for identification and classification of cells.

## Abstract 2

A microfluidic-based *in situ* single-cell recognition system (ISCRS) to analyze phosphatidylcholines for the cells classification.

## Graphical abstract

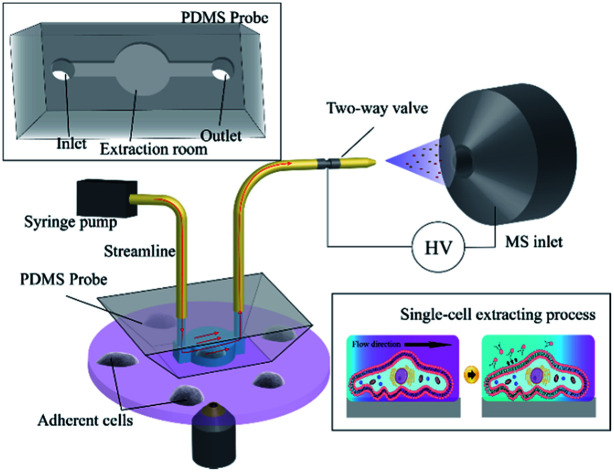

Cell analysis unveils life's mysteries and provides fundamental information for disease therapy.^1^ Identification of a cell type as well as its contents are key points in life sciences. In conventional methods, cells were identified in population,^2^ where we could obtain enough signals to read the information in the cells. Obviously, the obtained results are the average of a cell population.^3^ However, at a single-cell resolution cells with identical genes usually express inhomogeneous characteristics such as membrane components, metabolites and protein expression levels.^4^ Therefore, obtaining the information from individual cells of a cellular population is of major concern.^5^

Single-cell analysis has attracted considerable attention in recent years.^4*a*,6^ A more accurate representation of a tissue can be built through the analysis of metabolites, protein, or phospholipid contents of an individual cell.^1*a*,7^ Therefore, various approaches such as microfluidics,^8^ optical tweezers,^9^ fluidic force microscopy,^10^ and single-cell mass spectrometry^11^ have been utilized for single-cell analysis. Most of these techniques require fluorescent labelling of each cell, which limits their applications for the analysis of unknown cells or unknown components of a cell.^12^ Mass spectrometry (MS) is a powerful tool for single-cell analysis,^13^ because of its high sensitivity and ability to analyze multi-compound simultaneously.^14^ MS analysis of a single cell in suspension or droplets has been demonstrated.^15^ However, signals from a cells' suspension are not representative of the cells in its natural growing environment. Several methods such as single-probe or live-MS have their innate advantages to analyze intracellular contents.^16^ Yet, intracellular contents fail to reflect a whole cell which including significant information such as lipids in cytomembrane. On the contrary, nanospray desorption electrospray ionization mass spectrometry (nano-DESI) can analyze surface contents of a cell.^17^ However, part of intracellular contents could be missed applying this approach. In view of this, lipid analysis from a whole single-cell in adherent culture through MS remains a challenge.

In the present work, we have developed a microfluidic-based *in situ* single-cell recognition system (ISCRS, Fig. S1, ESI†) to extract a single-adhered-cell and analyze its phosphatidylcholine (PC) compositions through MS. This system performed well for the isolation of a whole cell from culture medium which excludes the signal interference of the solution composition. In addition, whole-cell extraction was realized for *in situ* online analysis. This could be a potential tool for understanding the cellular heterogeneity. The U87-MG cells (U87), human hepatoma (HepG2) cells, human epithelial colorectal adenocarcinoma (Caco-2) cells and human umbilical vein endothelial cells (HUVEC) were extracted for the single-cell identification and classification.

The ISCRS was consists of a flow injection, observation, operation, and detection system (Fig. S1 and S2, ESI†). In the flow injection system, a syringe pump was utilized to control the flow of solvent in the microfluidic channel for a cell's extraction. A microscope was used to visualize the capturing of cell under investigation in the microfluidic single-cell probe, which was stationed on a movable XYZ-stage. The probe, as the core component of the analytical system, was fabricated from polydimethylsiloxane (PDMS) by standard soft lithography techniques^18^ (Fig. S3 and S4, ESI†). The size and design were tested by a simulation using the COMSOL Multiphysics software to comprehensively understand the flow properties and the working area. Dead volume was decreased to a minimum limit to provide a uniform velocity distribution in the working plane (Fig. S5, ESI†). In each experiment, a target cell was first focused under the microscope of observation system (Fig. 1). Then the probe was adjusted and buttoned tightly on the Petri dish to capture a single cell in the extraction compartment. Due to the elasticity and tightness of the PDMS probe (Fig. 1a), the target cell was completed isolated from the culture environment and prevented the interference from the neighbour cells. Subsequently, the methanol as a solvent was introduced for the single-cell extraction. Methanol was used, because of its capability for the efficient extraction of PC in the cytomembrane and inside the cell, and compatibility for electrospray ionization. Finally, the extraction solution was analyzed by an electrospray quadrupole time of flight mass spectrometer (ESI-QTOF-MS). Because of methanol, the protein on the surface of the cell membrane was inactive and lost their selective permeable property. In each extracting process, membrane lipids accompanied by dissociative lipids in cytoplasm and organelle lipids are all extracted by the solvent. As a result, the PC analysis from a whole cell was achieved (Fig. 1b).

**Fig. 1:**
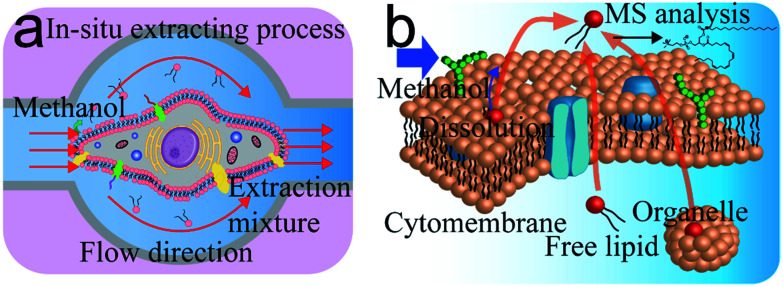
Mechanism of single-cell extraction and analysis based on *in situ* single-cell recognition system (ISCRS). (a) Illustration of the *in situ* single-cell extracting process. (b) Mechanism of procedure for PC compounds analysis.

The detectable signals from a single cell (MCF-7 as a model cell) were then analyzed (Fig. 2). At beginning, a target cell was captured by the observation and operation system and then was isolated from its environment by the single-cell probe (Fig. 2a). The flow rate was set at 0.4 μL min^−1^ to inject methanol as the extraction solution. Base peak chromatograms (BPC) of several PC compounds (confirmed by lipid maps and MS/MS spectra in Fig. S6, ESI†) were recorded to monitor the whole extracting process (Fig. 2b). The whole process from methanol injection to a cell extraction required 12 min. The background signal was observed for the first 5 min, which was due to the flow process of the extracted compounds from the extraction room to the MS detector. The connection volume has consisted of the channel volume (50 μm inner diameter and 15 cm long) and necessary connection part (approximately 2 μL), which lead to the calculated blank time of 5.7 min. The broadened error range of the detection time was attributed to the diffusion of a cell's contents during the transmitting procedure. Thus, the actual extracting time was ∼6–7 min in the chromatogram. The volume of the extraction room was 0.39 nL. Therefore, the static extraction ratio of a cell was 1 : 130 (average volume of a cell at 3 pL). However, due to fluid flow the dynamic extraction ratio was much lower than the static one. The value could be calculated from Fig. 2b which was approximately 1 : 1364. The process of a cell's extraction was recorded (Video S1, ESI†). The extracted cell was fixed on the culture dish due to the dehydration of methanol. The MS spectrum (Fig. 2c) also exhibited several peaks corresponding to PDMS, which was resulted from the incomplete crosslinks of the monomer in the polymer (pre-treatment of the PDMS probe by a series of solvents can alleviate this phenomenon). For better identification of the cell qualitatively, ten authenticated PC compounds were analyzed as shown in Fig. 2d. The relative intensities were summarized as the identity code of this cell (Table 1).

**Fig. 2:**
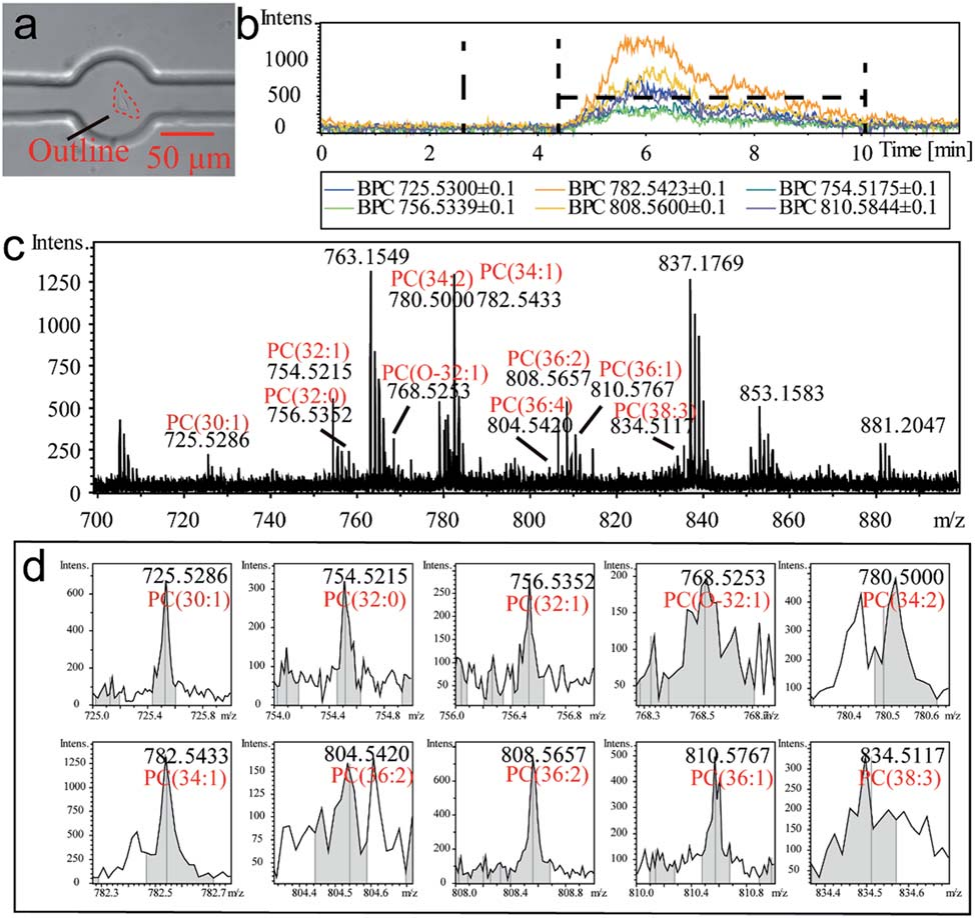
Single-cell extraction process and monitor of online extracted components analysis by MS. (a) Micrograph of an extracted MCF-7 cell. (b) BPC of several PC compounds at the period of whole single-cell extraction. (c) MS result of the extracted components from an MCF-7 cell. (d) MS peak from different species of PC compounds.

**Table 1:** Identified phosphatidylcholine in an MCF-7 cell

Compound	Formula	Ionic formula	*m*/*z*	Intensity
PC (30 : 1)	C_38_H_74_NO_8_P	[M + Na^+^]	725.5286	690
PC (32 : 1)	C_40_H_78_NO_8_P	[M + Na^+^]	754.5215	325
PC (32 : 0)	C_40_H_80_NO_8_P	[M + Na^+^]	756.5352	289
PC (O-32 : 1)	C_40_H_76_NO_9_P	[M + Na^+^]	768.5253	195
PC (34 : 2)	C_42_H_80_NO_8_P	[M + Na^+^]	780.5000	491
PC (34 : 1)	C_42_H_82_NO_8_P	[M + Na^+^]	782.5433	1260
PC (36 : 4)	C_44_H_80_NO_8_P	[M + Na^+^]	804.5420	158
PC (36 : 2)	C_44_H_84_NO_8_P	[M + Na^+^]	808.5657	755
PC (36 : 1)	C_44_H_86_NO_8_P	[M + Na^+^]	810.5767	504
PC (38 : 3)	C_46_H_82_NO_8_P	[M + Na^+^]	834.5117	343

According to the approach above, four cell lines including Caco-2, HUVEC, MCF-7 and U87 cells were tested for the identification and classification of different cells. Totally 98 cells from four cell lines were extracted and summarized for their identity code (Fig. S7–S18, ESI†). The extracting process of every single cell was similar and showed little difference in extraction time. However, the MS results were variant which revealed single-cell heterogeneity in phospholipids expressing in its population. The regularity of PC expressing in different cell lines was displayed (Fig. 3). The result could display the PC abundance by box height and reveal single-cell heterogeneity by dispersion of the distribution. This indicated that the majority of cells showed little difference in the content of PC (34 : 1), which was the highest PC compound in the cell membrane. However, other compounds showed variation in terms of abundance and distribution range such as PC (32 : 1), PC (36 : 1) and PC (36 : 2). These PC compounds could act as key molecules in a cell which could reveal different character of each cell line. For instance, based on the *t*-test, the PC (36 : 1) was in more abundance than PC (36 : 2) in U87 cell line (Fig. 3a). While this difference was not significant in other cell lines (Fig. 3b–d). The abundance of PC (38 : 3) in U87 was obviously higher than in the other cell lines, and also showed more individual variation. This could be significant information to distinguish U87 cells among other cells. Linear discriminant analysis (LDA) approach was applied, to comprehensively measure the population difference in PC expression and establish a method for cell identification. In this approach, all the data of individuals from four human cell lines were mixed together with the information of cell names, numbers of cells, and identity code. After the dimensionality reduction and linear discriminant calculation, the result was displayed in Fig. 3e. Two factors were calculated as the most suitable dimension for the classification. And 98 individuals were totally mapped on the scatter plot. It showed that each human cell line occupied a single group. The group distances indicated resolving abilities between different human cell lines. Single-cell heterogeneity could be the main influence factor for the inaccuracy of cell classification. There was little contact ratio between every group and the accuracy rate of classification was 91.8%. These results suggest the competency of this classification method for an effective identification of a single cell.

**Fig. 3:**
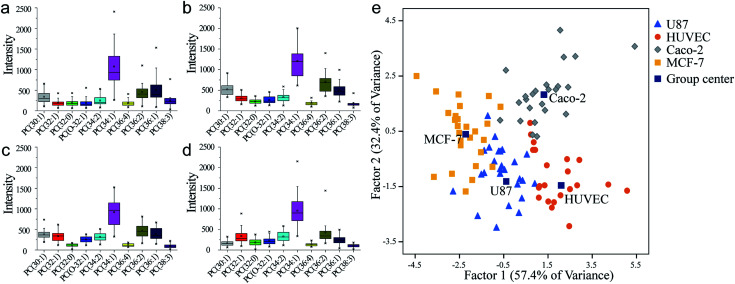
PC analysis of each human cell line in same cultural condition. (a) U87. (b) HUVEC. (c) Caco-2. (d) MCF-7. (e) Classification by different human cell lines by LDA.

## Conclusions

In conclusion, we have established a methodology for phosphatidylcholine detection from whole-cell extraction at a single-cell resolution that could evaluate the heterogeneity inside a human cell line. This could be a precise evaluation result for the detection of differentiated individuals in a cell population. Moreover, the approach was able to accomplish the single-cell classification of human cell lines by phospholipids expression. Although single-cell heterogeneity had a strong influence on classification, making it difficult to strictly identify one cell through a complex and mixed sample. Future prospects could be focused on improving experiment throughput and optimizing device's sensitivity. As the increasing of standard samples and more accurate detection of an individual, more precision classification would be realized, which might support a sufficient and effective tool to distinguish different types of cells through phospholipids. As a result, the approach opened a potential way for the label-free cell identification and auxiliary disease diagnosis.

## Conflicts of interest

There are no conflicts to declare.

## Supplementary Material

SC-011-C9SC05143K-s001

SC-011-C9SC05143K-s002

SC-011-C9SC05143K-s003
