# Supplementary material for: Single-cell identification by microfluidic-based in situ extracting and online mass spectrometric analysis of phospholipids expression
Source: Chem Sci. 2019 Nov 11;11(1):253–6. doi: 10.1039/c9sc05143k (PMC8132990; doi:10.1039/c9sc05143k)
Supplement: SC-011-C9SC05143K-s003 [file SC-011-C9SC05143K-s003.pdf]

Electronic Supplementary Material (ESI) for Chemical Science.
This journal is © The Royal Society of Chemistry 2019

Supporting Information (SI) for

Single-Cell Identification by Microfluidic Based In-situ Extracting and Online Mass Spectrometric Analysis of Phospholipids Expression

Qiushi Huang+, Sifeng Mao+, Mashooq Khan, Weiwei Li, Qiang Zhang, and Jin-Ming Lin*

Department of Chemistry, Beijing Key Laboratory of Micronalytical Methods and Instrumentation, MOE Key Laboratory of Bioorganic Phosphorus Chemistry & Chemical Biology, Tsinghua University, Beijing, 100084, China

Contents

1. Fabrication of the in-situ single-cell recognition system (ISCRS)................................3

2. Design and fabrication of the single-cell probe...6

3. Numeric simulation 8

4. Cell culture and sample preparation 10

5. MS/MS spectra confirming the lipid classes 11

6. MS spectra of individuals from different cell lines...13

1. Fabrication of the in-situ single-cell recognition system (ISCRS)

The ISCRS contained a flow injection system, an observation and operation system and a detection system(Figure S1).

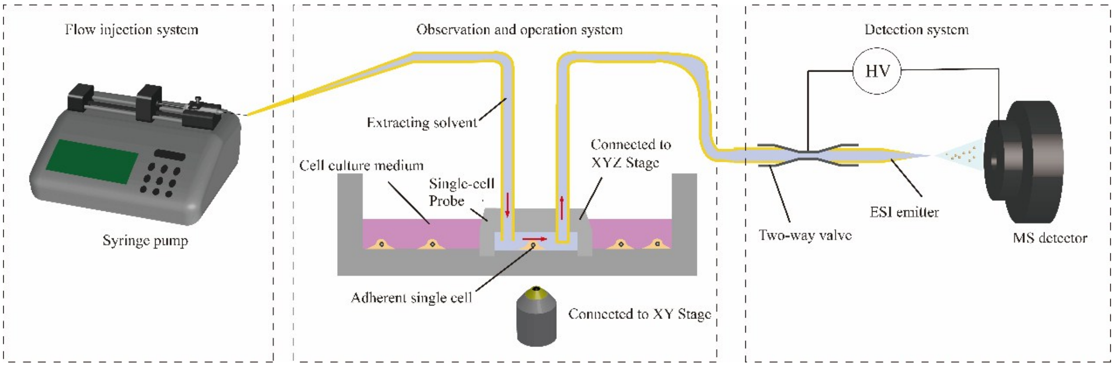

Figure S1. Schematic diagram of the ISCRS.

In the flow injection system, a gas tight syringe (Hamilton, Graubunden, Switzerland) was connected to the inlet of the single-cell probe by capillary with 50 μm inner diameter and 363 μm outer diameter with 20 μm standard polyimide coating，which was purchased from Innosep (11 Changchun Road, Zhengzhou High New-Tech Zone, China). The syringe for flow injection were driven by a syringe pump (Hamilton, Pennsylvania, USA).

In the positioning system, cell samples were placed on an XY stage of an inverted microscope (Leica DMI 4000 B, Wetzlar, Germany). An XYZ stage (Sigma KOKI Co., Ltd.) served as the device holder for postioning the single-cell probe to desirable point and finish the single-cell capturing by lift and fall down the device.

In the detection system, the other aperture of the single-cell probe were connected to a commercially available electron spray ionization (ESI) device by the same capillary in flow injection system. We used a home-made electro-emitter with the tip diameter of 20 μm. The detector was an electrospray quadruploe time of flight mass spectrometer (micrOTOF II, Bruker, Germany).

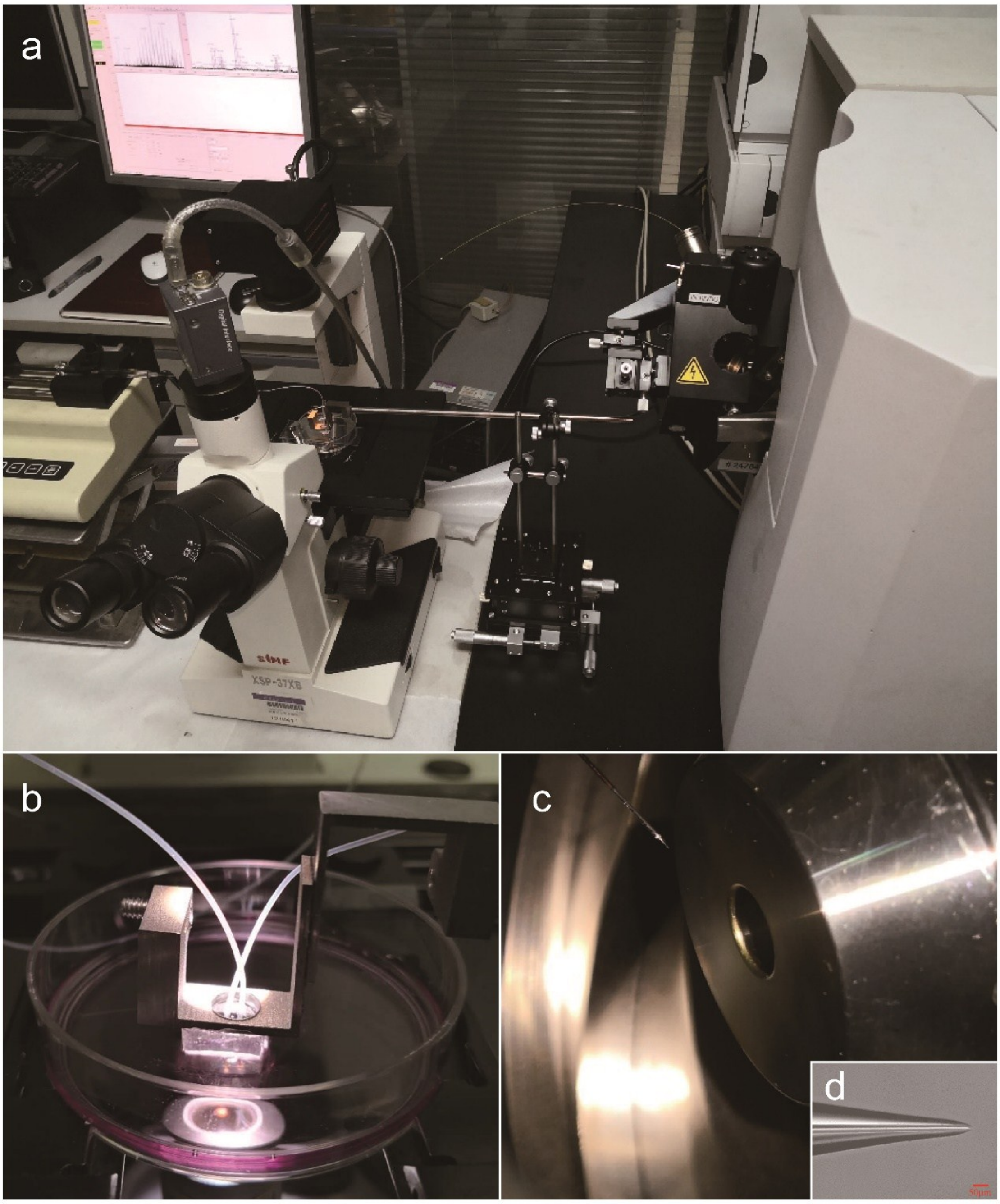

Figure S2. Photograph of the ISCRS. (a) Total view. (b) single-cell probe and its connection approach. (c) ESI emitter. (d) Tip of the ESI emitter.

In all of the experiments, the single-cell probe was maintained at its position while the postion of cell samples were controlled by the XY stage of the microscope. A petri dish (Corning, New York, USA) with cell sample, filled with cell culture medium, was placed on the XY stage of a microscope. Then, the single-cell probe was immersed in

the cell culture medium and placed perpendicular to the surface of petri dish with a centain gap by the XYZ stage. The XYZ stage was functioned as a positioner to locate the single-cell probe and attach the probe to the petri dish. Due to the elasticity of the PDMS material, the probe was able to closely attach to the surface which generated an isolation area for the single-cell extraction inside the probe. After the single-cell probe was located on the target cell, the syringe pump was switched on and provided dynamics for the flow injection and MS analysis. The flow rate was set at 0.4 μL/min and the whole progress might consume 15 minutes from starting injection to ending extraction.

2. **Design and fabrication of the single-cell probe**

The probe was fabricated from polydimethylsiloxane (PDMS, Sylgard 184, Dow corning) by standard soft lithography techniques.

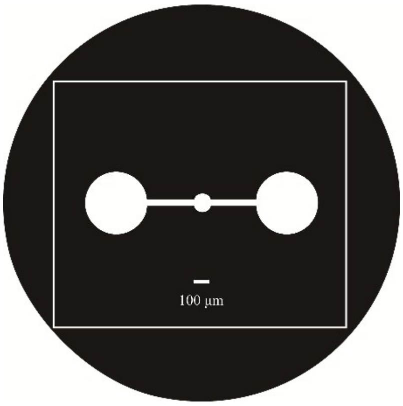

Figure S3. Mask design for single-cell probe.

The layout of the device was fabricated by coating photoresist SU-8 2050 (Microchem, Newton, MA, USA) on a silicon wafer. After photolithographic patterning (Figure S3), a mold was generated. Premixed 10:1 ratio of PDMS prepolymer and curing agent was poured onto the mold and cured. The cured PDMS was treated by following steps to decrease uncrosslinked oligomers and impurity: (1) Soaking in 100ml diisopropylamine at room temperature for 2 hours. (2) Soaking in 100ml toluene at room temperature for 2 hours. (3) Soaking in 100ml acetone at room temperature for 2 hours. (4) Dry at 70 ℃ for 12 hours and ready for use.

The width and height of the channel were about 50 μm (Figure S4a). The cell extraction room was fabricated into a cylinder with the width of 100 μm (Figure S4b). Two apertures for the fluid injection and outlet were punched by a syringe needle (Figure S4c). The final model of the main part was cut into a frustum of a pyramid (Figure S4d).

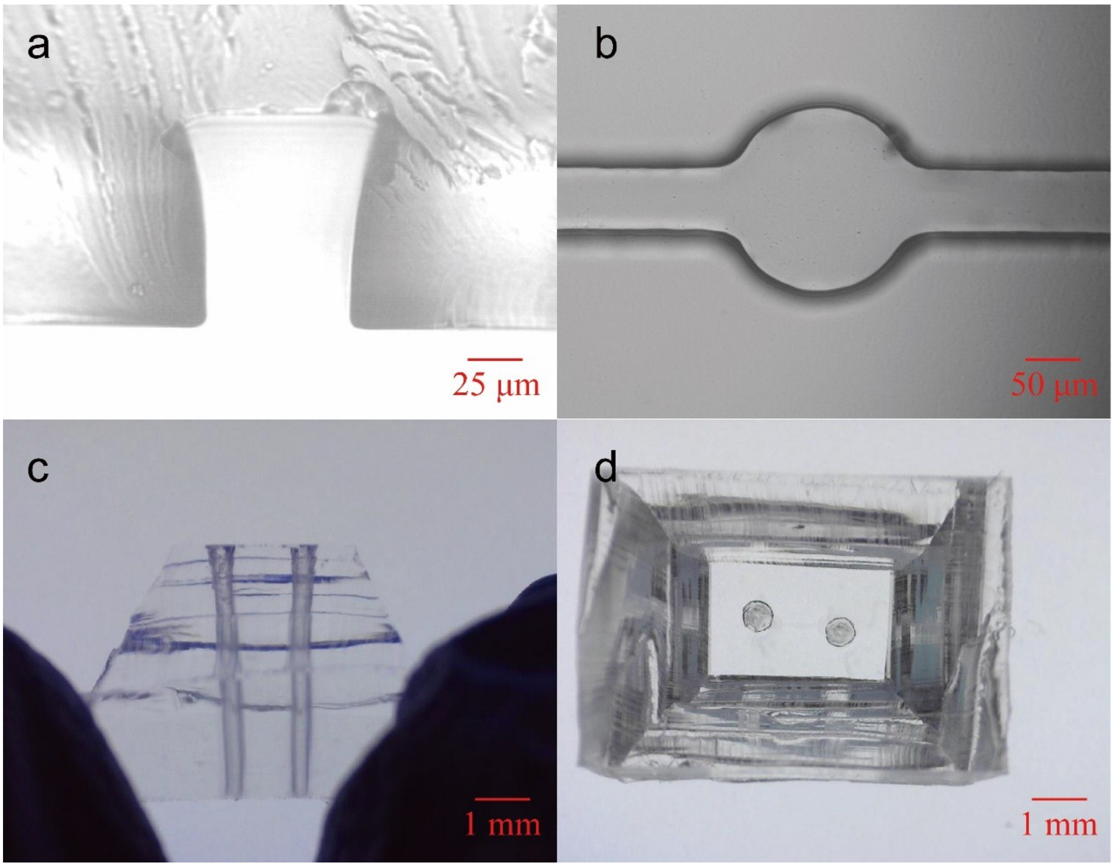

Figure S4. Single-cell probe. (a) Cross section of the channel. (b) Top view of the extraction room. (c) Two apertures for the fluid injection and outlet. (d) Top view of the probe.

3. Numeric simulation

COMSOL Multiphysics 5.3a (COMSOL) was used to carry out 3D simulations on a six-core, 64-bit computer (Dell) with 16 GB of RAM. The geometry of the single-cell probe was set as same as that in the experiment.

The width and height of the channel were 50 μm. The cell extraction room was set as a standard cylinder with the width of 100 μm. Injection flow rate was 0.4 μL/min. The injection solution was assumed to be water with a density of 999.7 kg/m^3 and a viscosity of 0.001 Pa·s. The simulations were run under steady-state conditions with the flow boundary conditions at the edges of the microfluidic chip probe perimeter sides set as open boundaries (equal to atmospheric pressure).

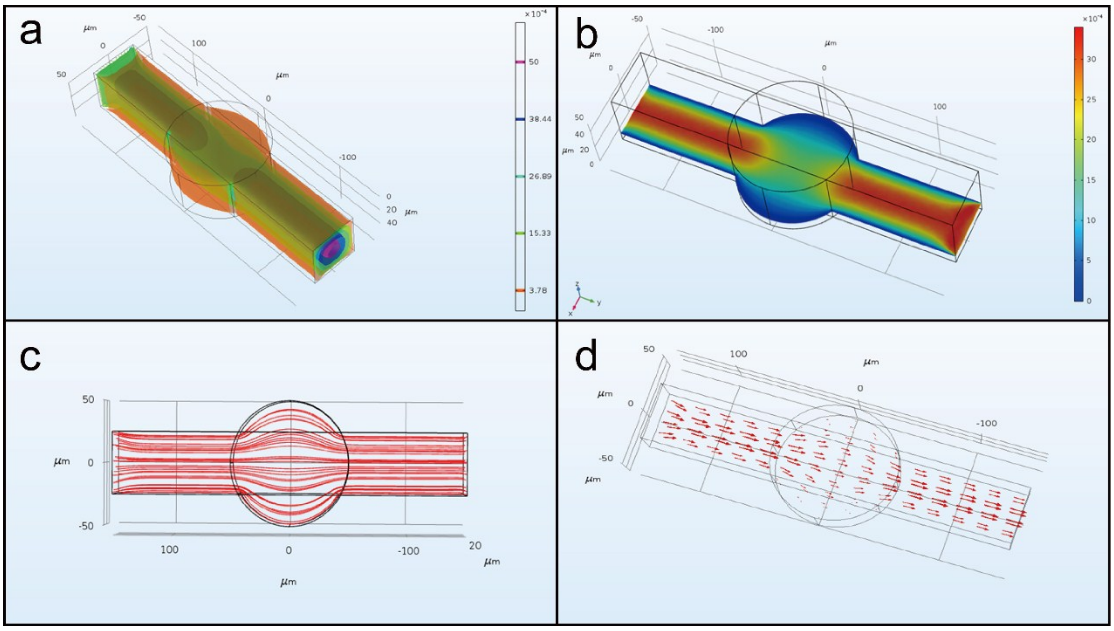

Figure S5. Calculated velocity in the simulation. (a) Velocity distributions of injection flow. (b) Velocity profile of the injection flow at the working plane which is at the same horizontal plane with the determinand. (c) Filament line of injection flow. (d) Flow distribution in the single-cell probe.

The velocity distributions of injection flow were shown in figure S5a. Due to the surface tension of solution and boundary friction, the flow velocity decreased as the distance increased form the boundary. However, the design of a circular extraction room helped to generate a uniform velocity distribution at the working plane (Figure

S5b). As long as the target cell was in the suitable working area, it would be immersed in a uniform flow for the single-cell extraction. Figure S5c showed the uniformity of filament line which indicated the stability of the device. It could be concluded that the flow velocity was higher in the channel than in the extraction room (Figure S5d). This would help to increase extraction time with the same volume of solution and decrease the solvent transit time in the channel.

4. **Cell culture and sample preparation**

U87 cells, Caco-2 cells, MCF-7 and HUVEC cells were purchased from Cancer Institute & Hospital of the Chinese Academy of Medical Science (Beijing, China). All cells were cultured in a humidified atmosphere of 95% air and 5% CO_2 at 37 °C. U87 cells were maintained in minimal essential medium (MEM, Corning, USA) with Earle’s Salts and L-glutamine supplemented with 10% fetal bovine serum (FBS, Corning, USA), nonessential amino acids, 100 units/mL penicillin, and 100 units/mL streptomycin. Caco-2 cells were maintained in RPMI-1640 (Corning, USA) with Earle’s Salts and L-glutamine supplemented with 10% FBS, nonessential amino acids, 100 units/mL penicillin, and 100 units/mL streptomycin. MCF-7 and HUVEC cells were maintained in Dulbecco’s modified Eagle’s minimal essential medium (DMEM, Corning, USA) with high glucose supplemented with 10% FBS, nonessential amino acids, 100 units/mL penicillin, and 100 units/mL streptomycin. All types of cells were maintained in Petri dishes for 2−3 days prior to commencing the experiments. All the experiments were carried out when the cells were in the exponential growth phase.

5. MS/MS spectra confirming the lipid classes

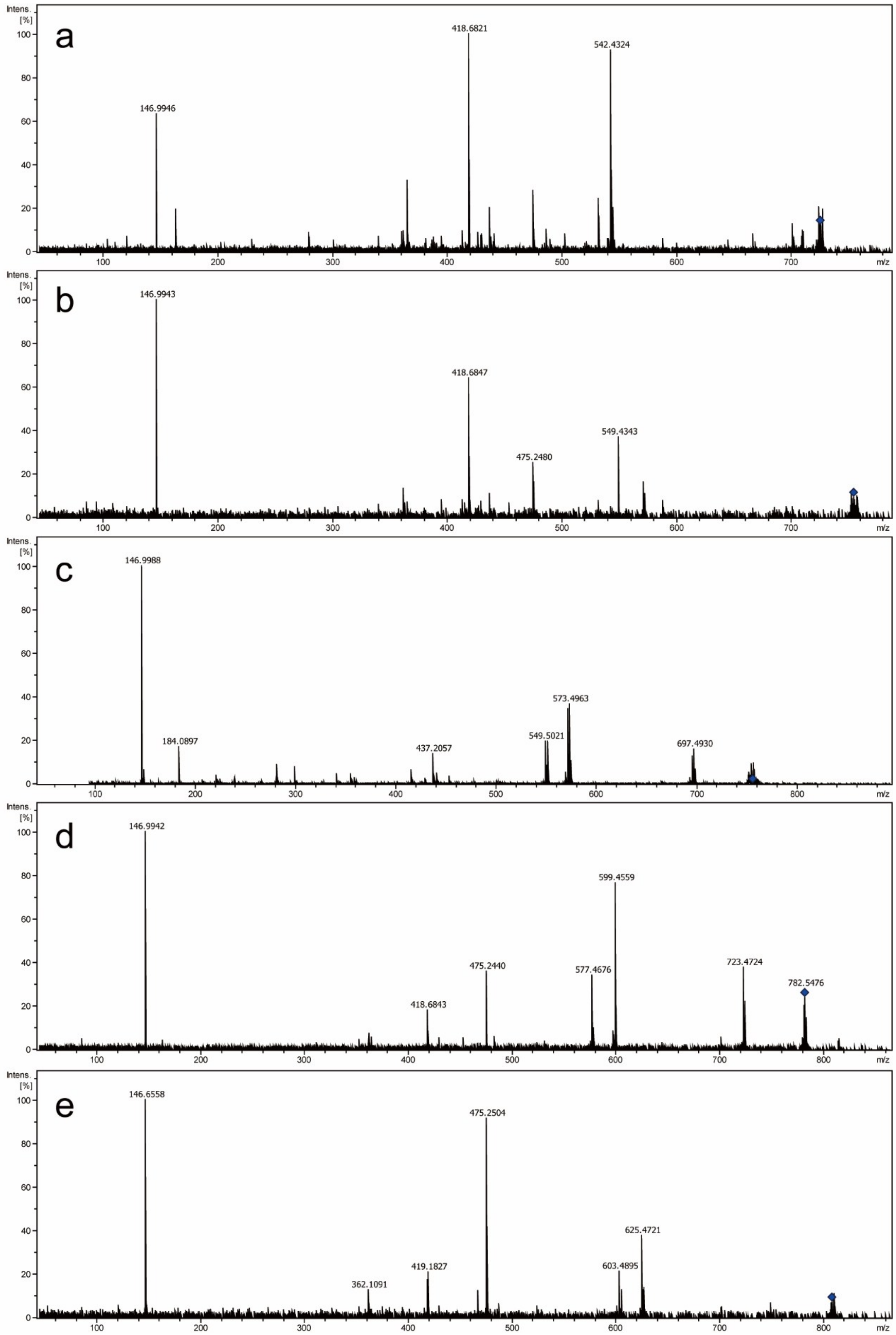

Figure S6 MS/MS spectra providing head group information to confirm the lipid classes. (a)PC (30:1) at M/z = 725.55. (b) PC (32:1) at M/z = 754.53. (c) PC (32:0) at

M/z = 756.54. (d) PC (34:1) at M/z = 782.56. (e) PC (30:2) at M/z = 808.57.

6. MS spectra of individuals from different cell lines

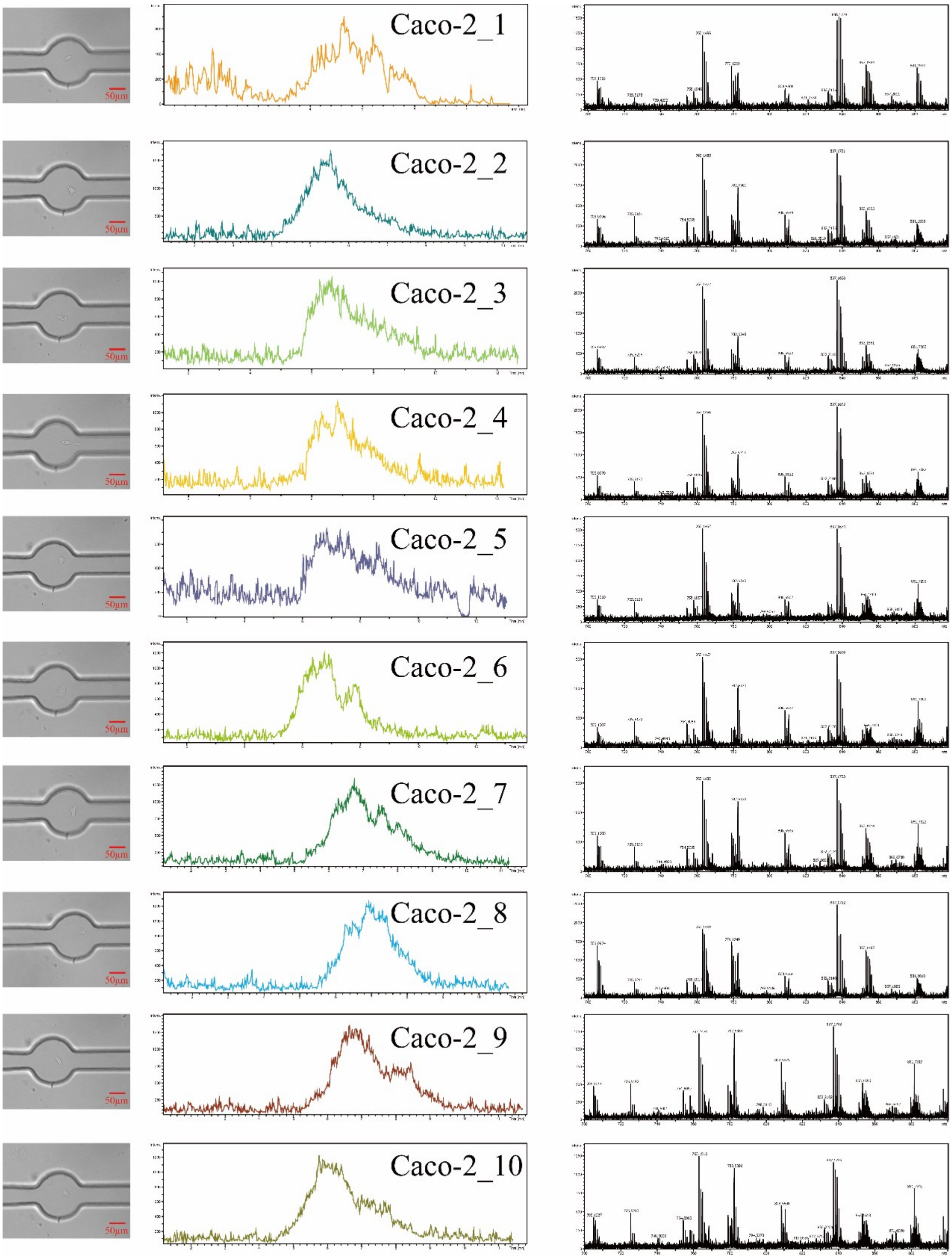

Figure S7. The microscopic image (left column), base peak chromatogram (middle column) and mass spectrum (right column) of the single Caco-2 cell from number 1-10.

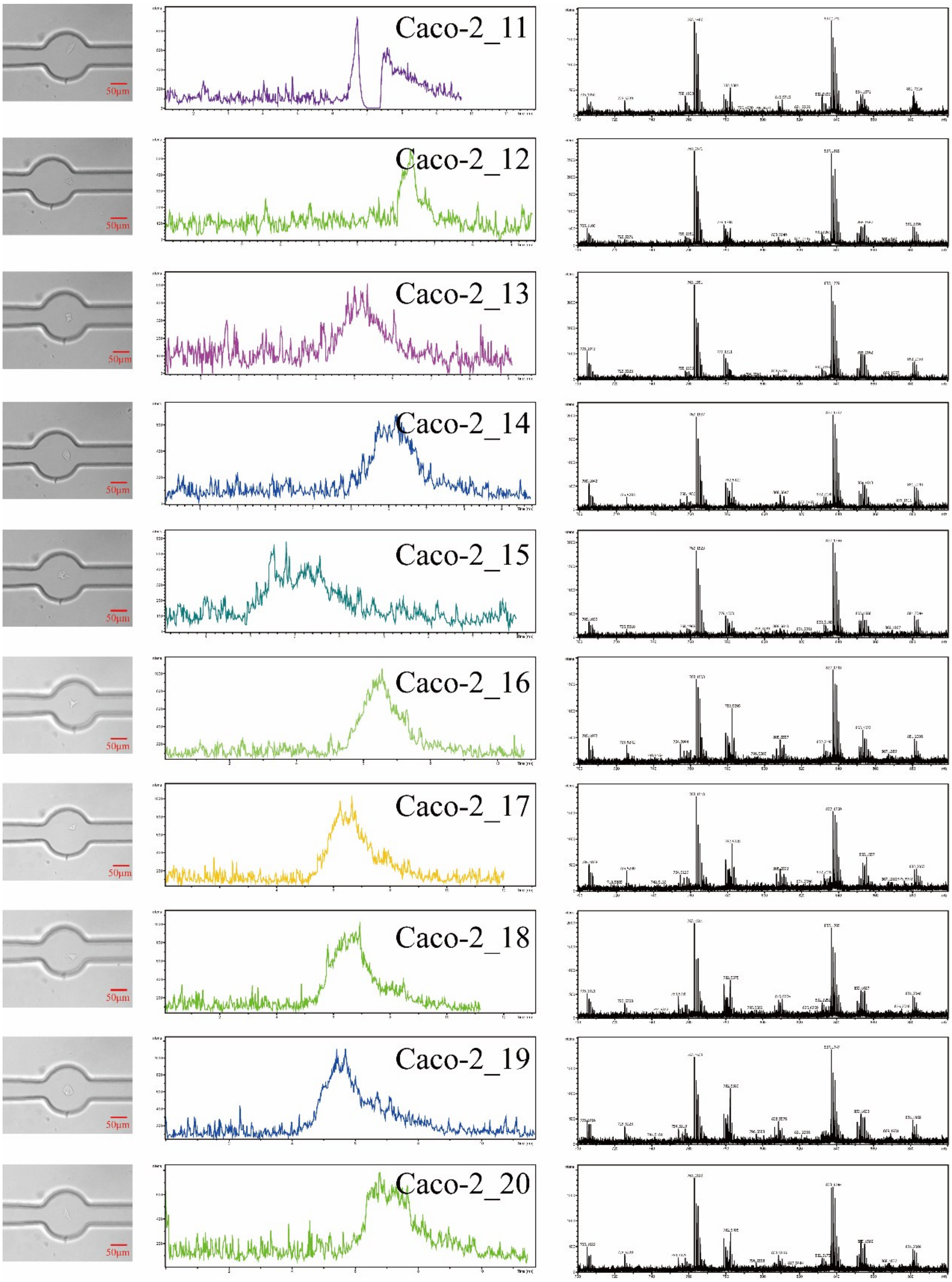

Figure S8. The microscopic image (left column), base peak chromatogram (middle column) and mass spectrum (right column) of the single Caco-2 cell from number 11-20.

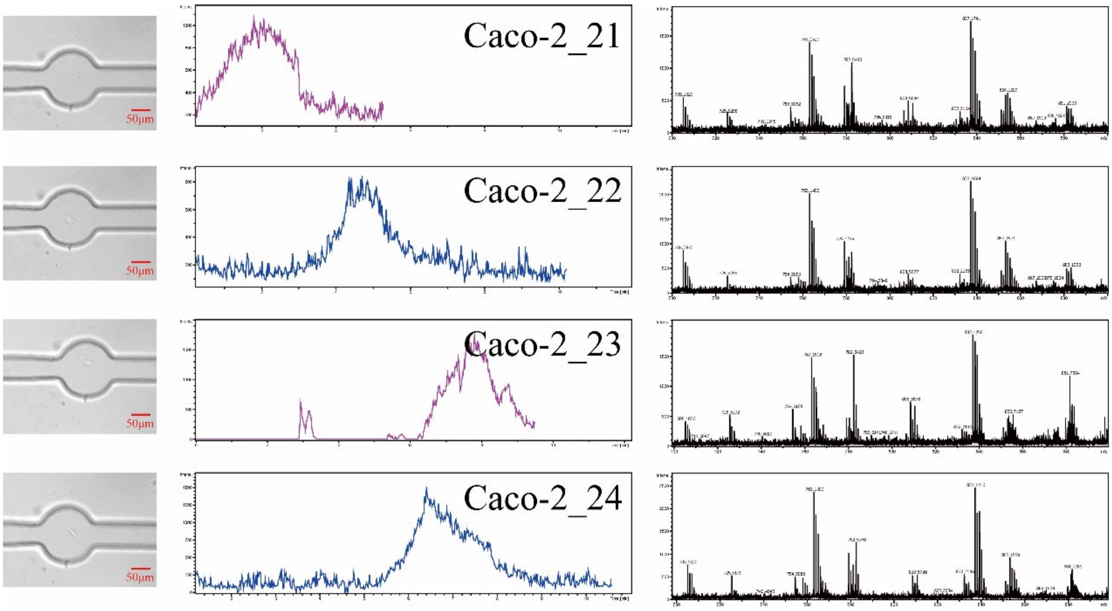

Figure S9. The microscopic image (left column), base peak chromatogram (middle column) and mass spectrum (right column) of the single Caco-2 cell from number 21-24.

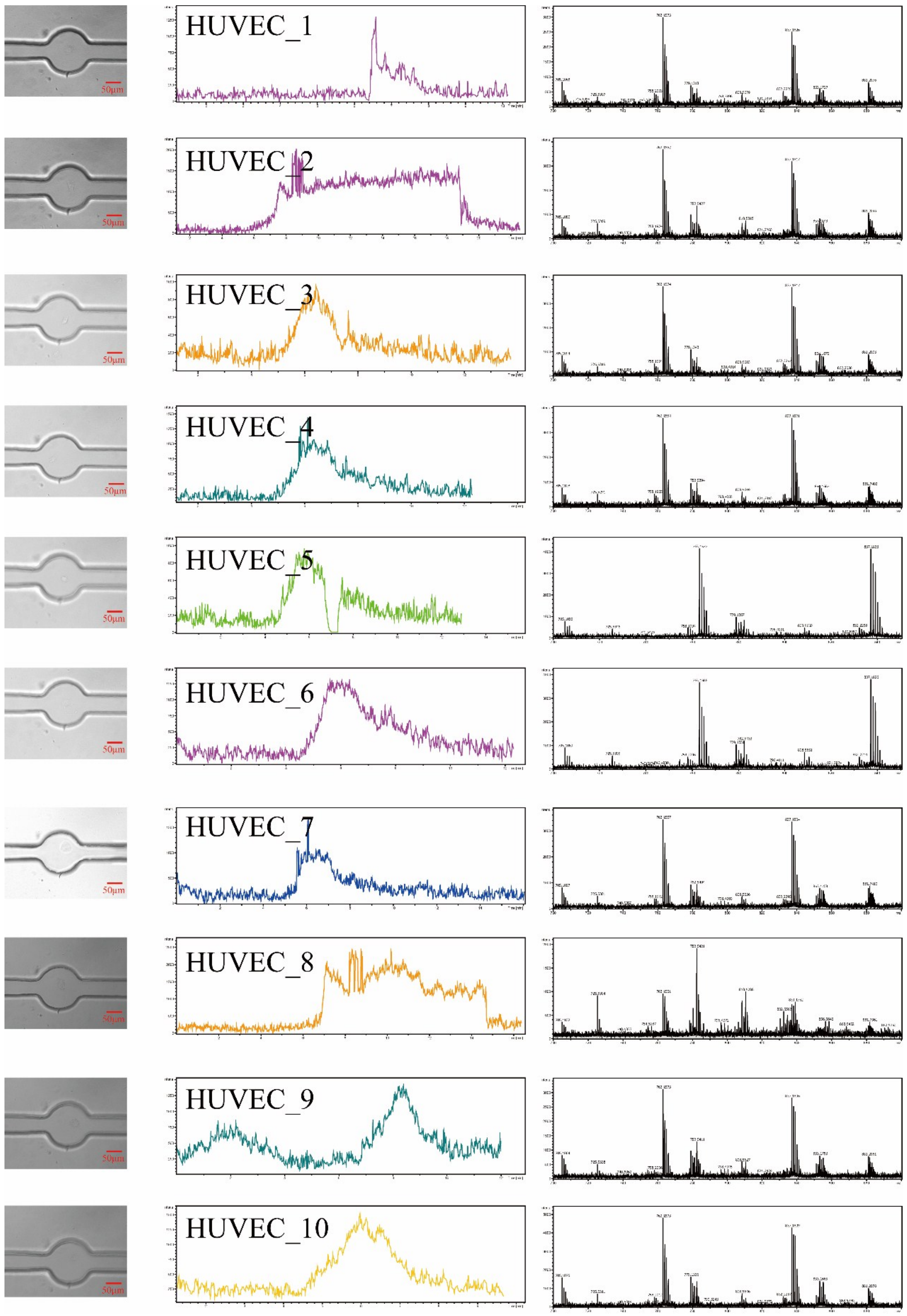

Figure S10. The microscopic image (left column), base peak chromatogram (middle column) and mass spectrum (right column) of the single HUVEC cell from number 1-10.

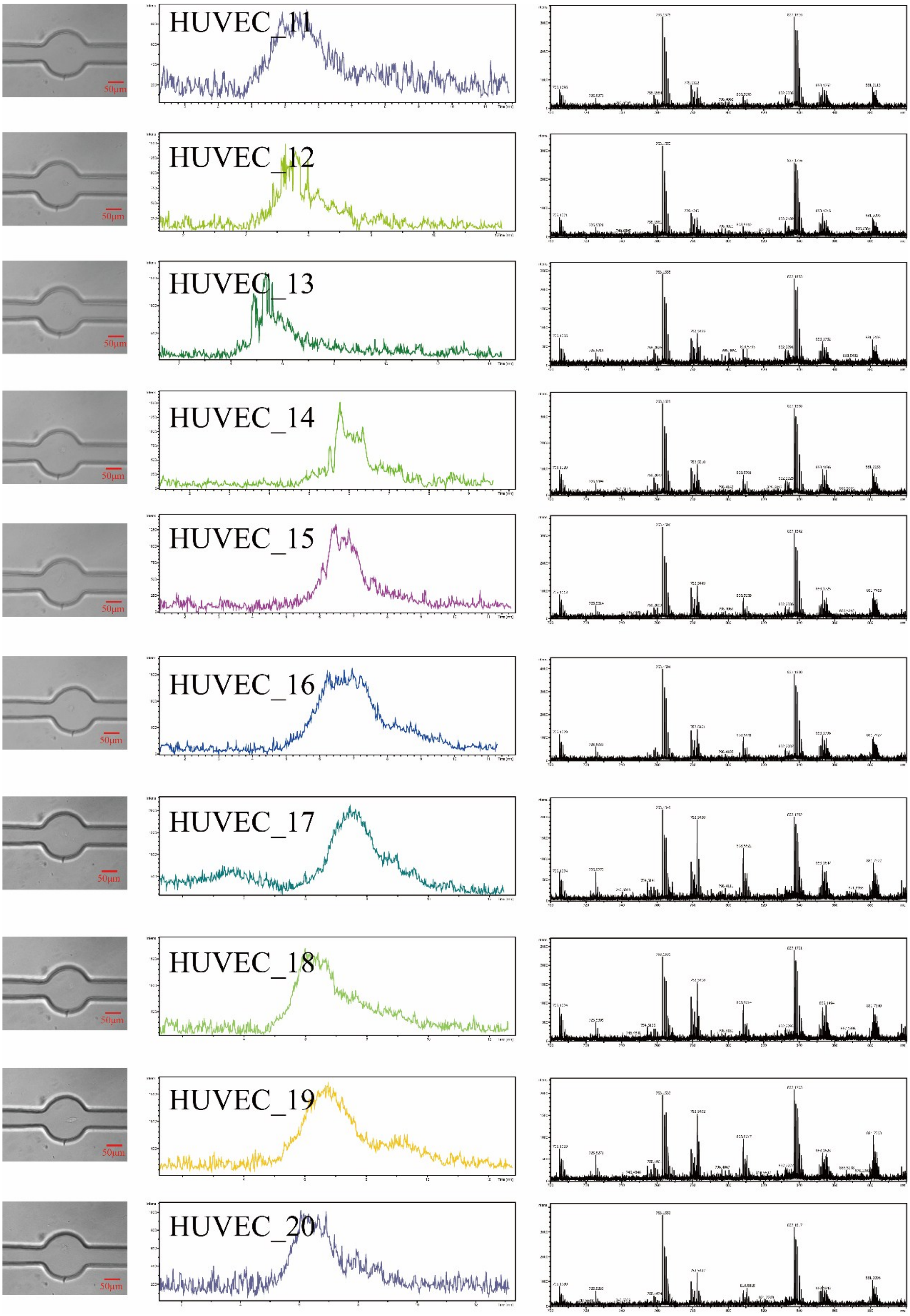

Figure S11. The microscopic image (left column), base peak chromatogram (middle column) and mass spectrum (right column) of the single HUVEC cell from number 11-20.

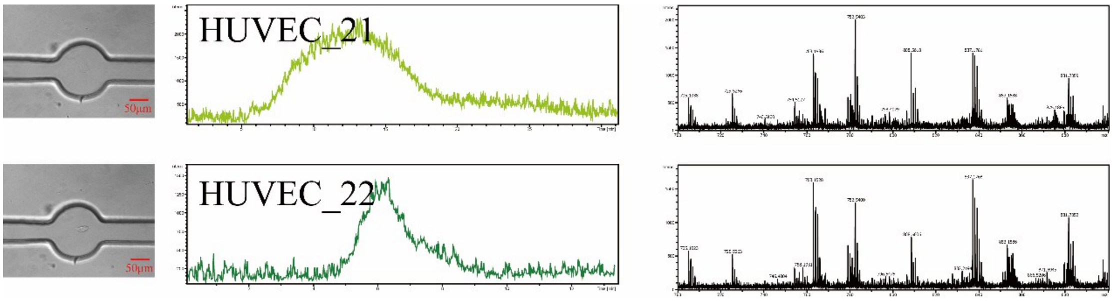

Figure S12. The microscopic image (left column), base peak chromatogram (middle column) and mass spectrum (right column) of the single HUVEC cell from number 21-22.

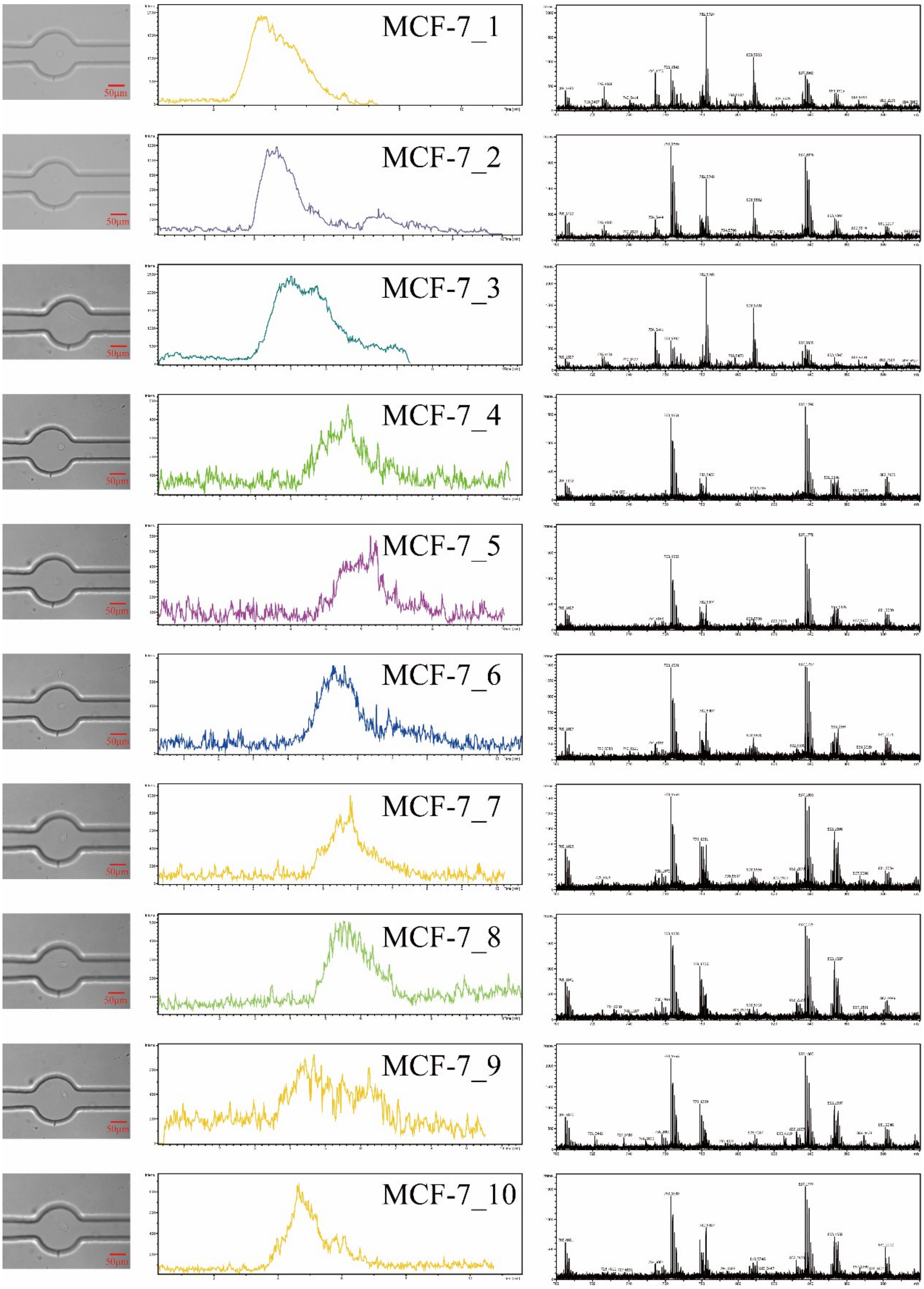

Figure S13. The microscopic image (left column), base peak chromatogram (middle column) and mass spectrum (right column) of the single MCF-7 cell from number 1-10.

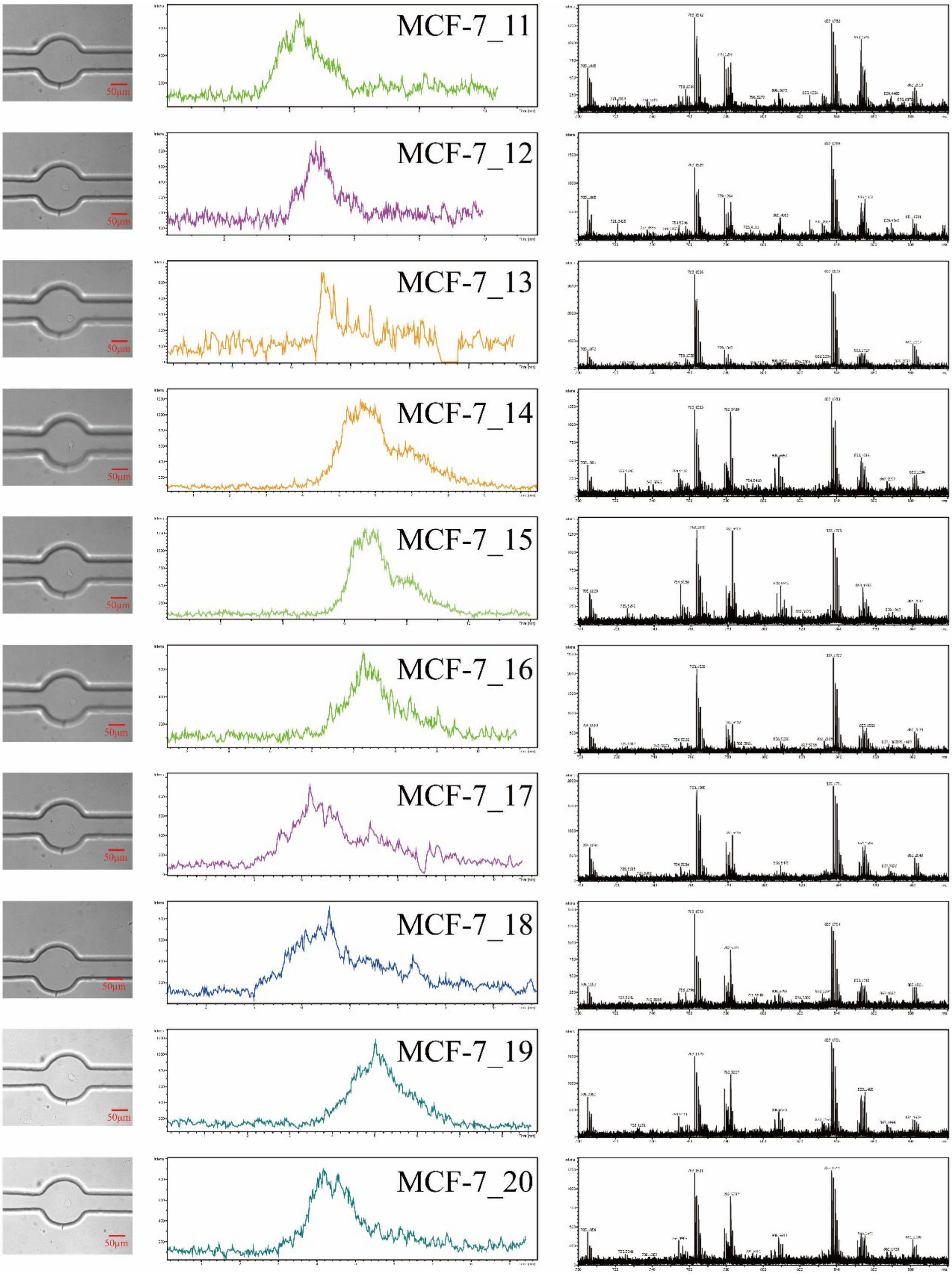

Figure S14. The microscopic image (left column), base peak chromatogram (middle column) and mass spectrum (right column) of the single MCF-7 cell from number 11-20.

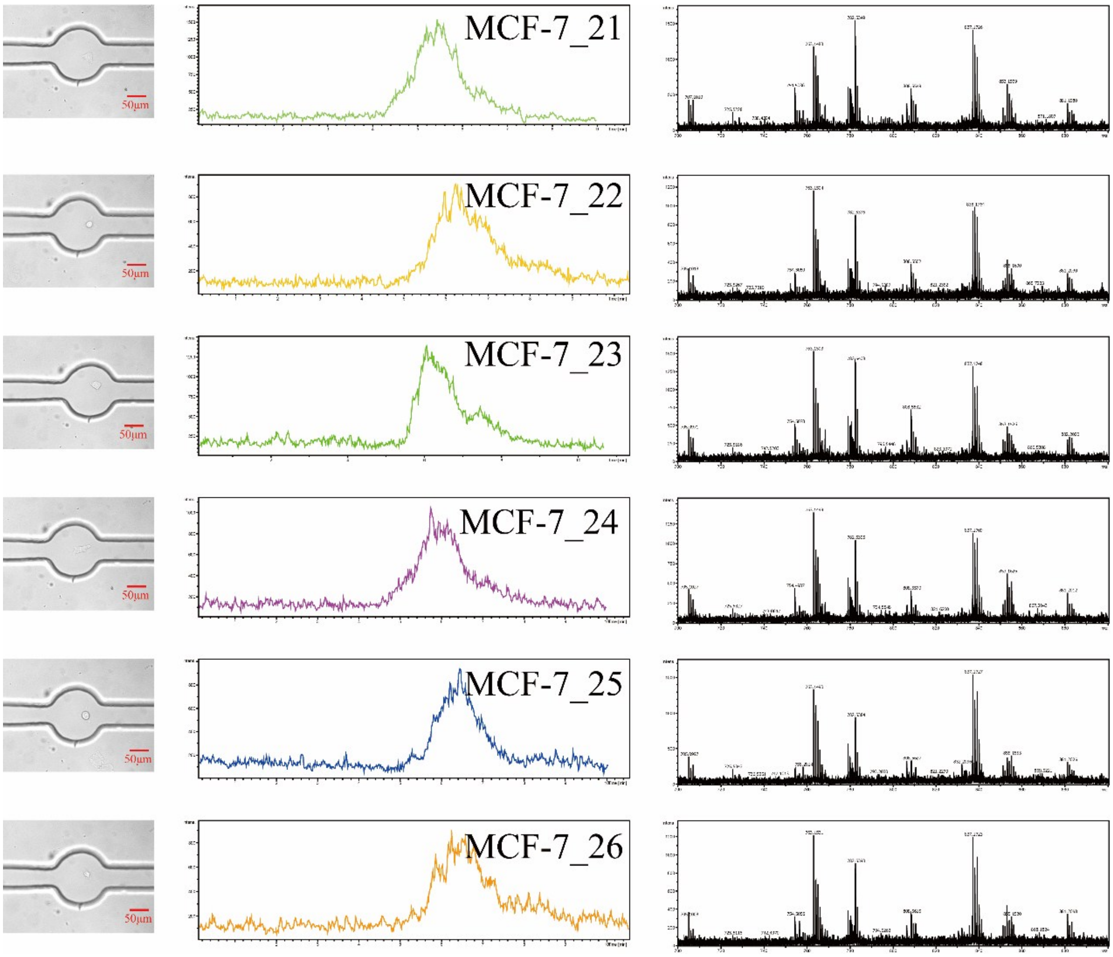

Figure S15. The microscopic image (left column), base peak chromatogram (middle column) and mass spectrum (right column) of the single MCF-7 cell from number 21-26.

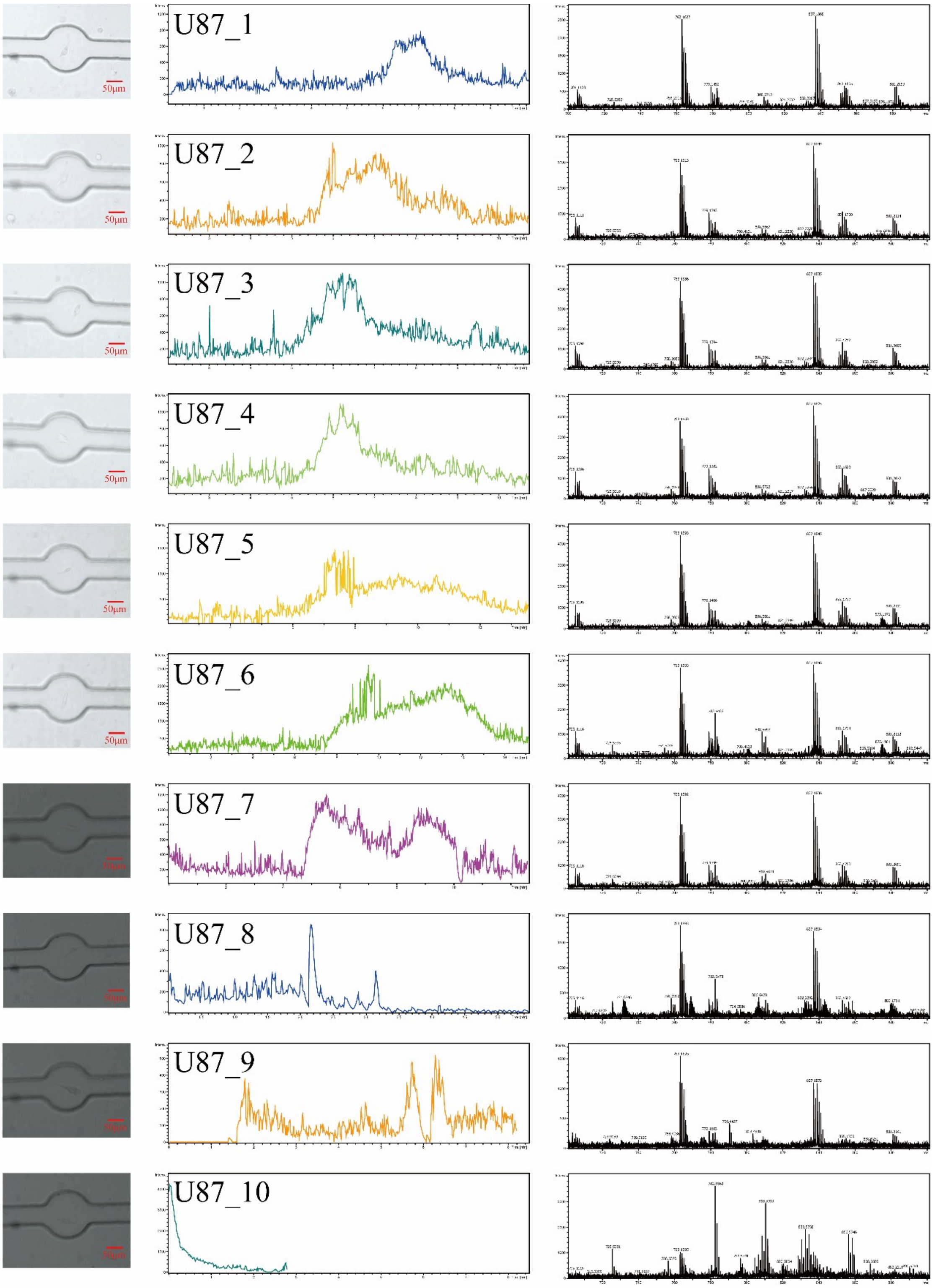

Figure S16. The microscopic image (left column), base peak chromatogram (middle column) and mass spectrum (right column) of the single U87 cell from number 1-10.

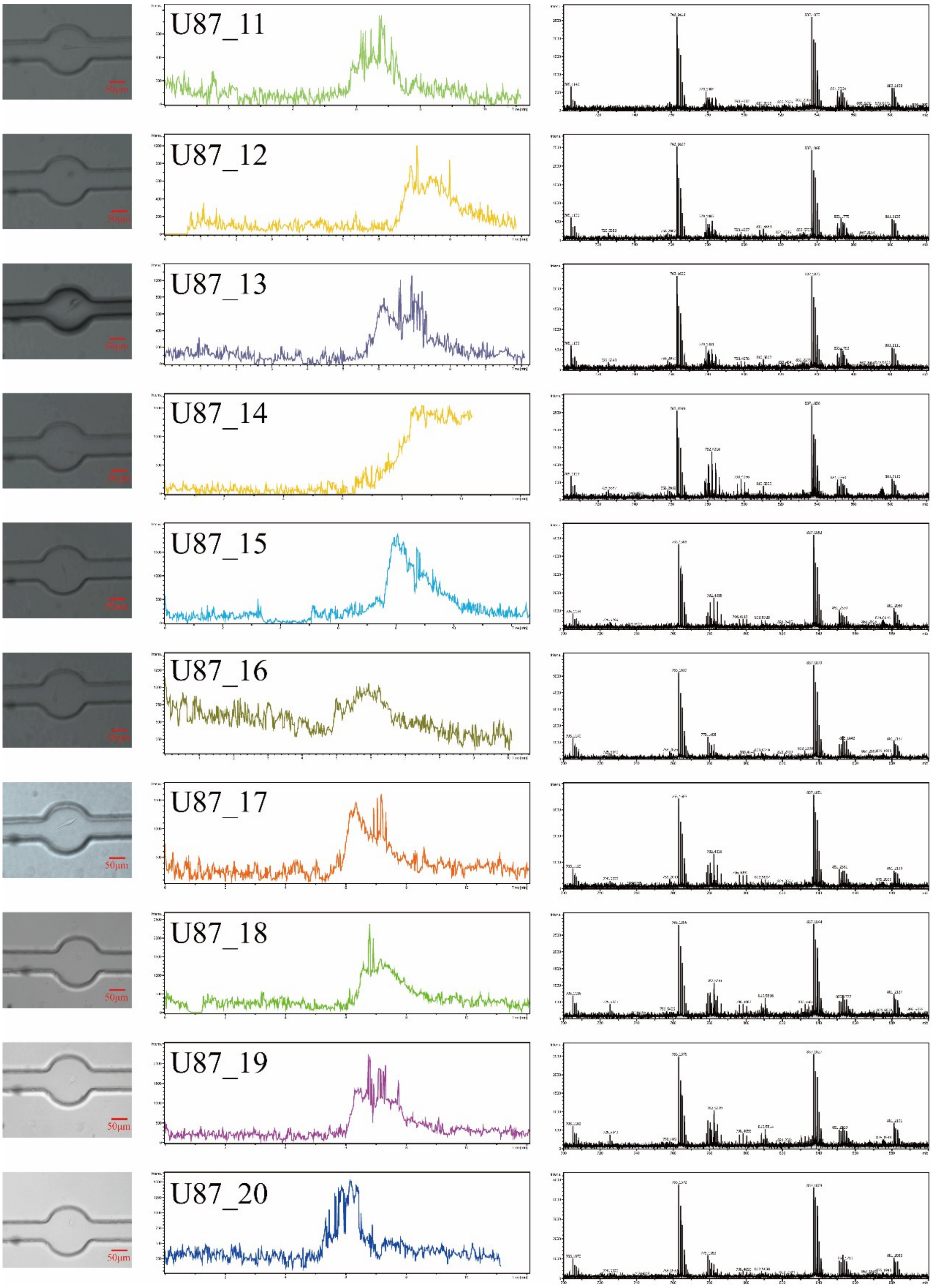

Figure S17. The microscopic image (left column), base peak chromatogram (middle column) and mass spectrum (right column) of the single U87 cell from number 11-20.

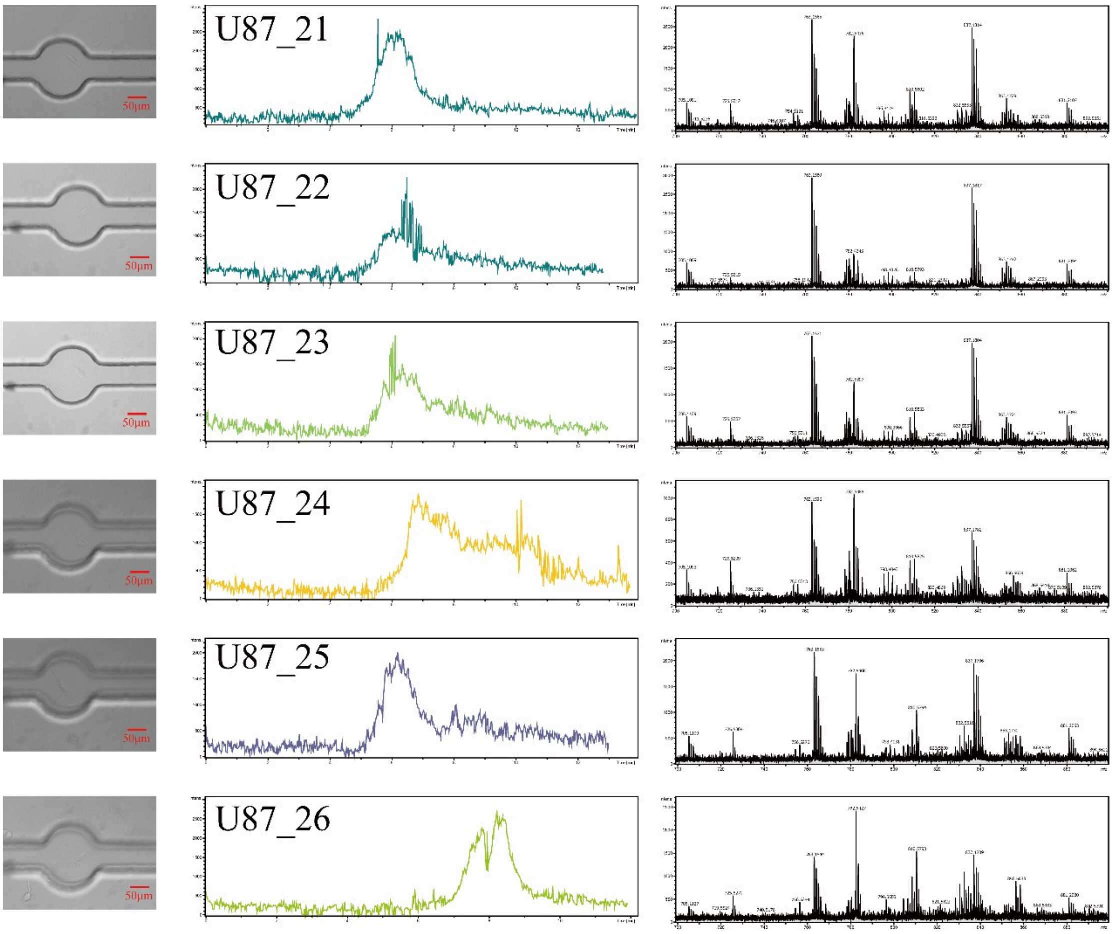

Figure S18. The microscopic image (left column), base peak chromatogram (middle column) and mass spectrum (right column) of the single U87 cell from number 21-26.

Supplementary Video S1 single-cell extraction

Supplementary Video S2 single-cell isolation
